# Social determinants and risk behaviors associated with prevalent Hepatitis C and HIV/HCV co-infection among male injection drug users in Nepal

**DOI:** 10.1186/s13690-017-0206-8

**Published:** 2017-09-04

**Authors:** Sampurna Kakchapati, Manju Maharjan, Bir Bahadhur Rawal, Sameer Mani Dixit

**Affiliations:** 1 Department of Mathematics and Computer Science, Faculty of Science and Technology, Prince of Songkla University, Pattani, Thailand; 2 Center for Research on Environment Health and Population Activities (CREHPA), Kathmandu, 44700 Lalitpur Nepal; 3 National Centre for AIDS and STD Control, Kathmandu, Nepal; 4 Center for Molecular Dynamics Nepal, Kathmandu, Nepal

**Keywords:** Hepatitis C, HIV, IBBS, Drug users, Nepal

## Abstract

**Background:**

Nepal is facing double burden of injecting drug use and HIV, yet the problem of Hepatitis C Virus (HCV) has not been so well addressed, where there is large population known to be at risk for HCV. This study assessed the prevalence of HCV infection and HIV/HCV co-infection among male injection drug users (IDUs) in Nepal and identified factors associated with infection.

**Methods:**

Cross-sectional surveys in 2015 aimed to sample 1045 male IDUs in the Kathmandu valley, Pokhara Valley and Eastern Terai districts of Nepal. Information about socio demographic characteristics, injecting and sexual risk behaviours were obtained, and biological specimens tested for HCV and HIV. The logistic regression model was used to identify the determinants associated with HCV and HIV/HCV co-infection.

**Results:**

HCV prevalence was 28.8% and HIV/HCV co-infection was 4%. Among the 6% of HIV positive male IDUs, 65% were found to be co-infected. The multivariate logistic analysis revealed that HCV prevalence was higher in Eastern Terai districts, longer duration of drug use and injecting drugs and presence of HIV. Similarly, HIV/HCV co-infection was associated with Eastern highway districts, older age and longer duration of injecting drugs.

**Conclusion:**

The factors strongly contributing to HCV and HIV/HCV co-infection was longer duration of injecting drugs. Highest HCV and HIV/HCV co-infection was found in Eastern Terai districts. Target health interventions need to be focused in Eastern Terai districts and IDUs with longer duration of injecting drugs for the prevention of HCV and HIV/HCV transmission.

## Background

Human Immunodeficiency Virus (HIV) and Hepatitis C virus (HCV) are a major public health concern among male injection drug users (IDUs) worldwide [1, 2]. An estimated 130–150 million people are chronically infected with HCV infection causing half million deaths and the majority of cases occurred in low and middle-income countries [1–3].

IDUs are disproportionately affected by the HCV epidemic and bear by far the greatest burden of HCV of any population [1, 2, 4]. Among the estimated 16 million IDUs globally, about 8 million suffered from chronic hepatitis [4]. Injecting drug use accounts for 67% of HCV infections [5]. Co-infection with HIV is prevalent among IDU [4–6] and in fact, HCV has become a leading cause of death among HIV-positive people. HCV/HIV co-infection can rapid the progression of HCV [7], and co-infected people have lesser life expectancy [8]. HIV/HCV co-infection complicates anti-retroviral therapy (ART) for treatment of HIV infection, as several anti-retroviral drugs are poorly tolerated by co-infected patients. Early detection of co-infection is optimal so that HCV treatment can be commenced before initiation of ART [9].

In Nepal, there are about that 50,000 people injected drugs and HIV and HCV are a major burden among IDUs in the region [10–12]. Although, HIV among male IDUs has significantly decreased from 68% in 2002 to 6.4% in 2015 in Kathmandu Valley, IDUs are still the most key affected population compared to other groups with respect to HIV infections [10–12]. Multiple studies provide evidence that HCV prevalence is rapidly increased among male IDUs [4–6]. In Nepal the recent study in 2015 showed that the prevalence rate of HCV among male IDUs was 41.9% [10]. Sharing needles/syringes between injecting partners and re-use of needles kept in public places retain high-risk behavior among IDUs [1, 2]. Moreover, risk factors include use of unsterilized medical equipment, the transfusion of unscreened blood and blood products and social or cultural practices comprising of body piercing, circumcision, and tattooing [1, 13–16]. Sexual behaviour such as unprotected sex with sexual partners is also considered as risk for HCV [14]. HCV prevalence was higher among persons with multiple sexual partners or risk for sexually transmitted diseases than persons with durable faithful relationship [17–19]. However the risk is also linked with rates of exposure to nonsexual sources of HCV, such as injection drug use or shared razors and toothbrushes [17].

Although studies revealed that social determinants and risk behaviours are linked with HCV infection and HIV/HCV co-infection globally [4–6, 9, 13–19], limited studies associated with HCV prevalence among IDUs were documented in Nepal. The aim of the study was to assess HCV prevalence and HIV/HCV co-infection and social and behavioural factors associated with HCV prevalence and HIV/HCV co-infection among male IDUs in Nepal using Integrated Biological and Behavioral Surveillance (IBBS) survey data. In Nepal, IBBS surveys are regularly conducted among male IDUs from 2002 to 2015 to understand the emerging trends of HIV and HIV related risk behaviours, while there was no routine surveillance for HCV infection. This recent IBBS surveys in 2015 assessed HCV prevalence among male IDUs in Eastern Terai districts, Kathmandu Valley and Pokhara Valley of Nepal for the first time.

## Methods

A cross sectional study was conducted in the Kathmandu Valley, Pokhara Valley and Eastern Terai districts by the National Centre for AIDS and STD Control (NCASC) in 2015. In this study IDUs were defined as males, aged 16 years and above using injecting illicit drugs for at least three months prior to the date of the survey. About 340 male IDUs were recruited from Kathmandu Valley, 345 recruited from Pokhara Valley and 360 recruited from Eastern Terai Districts. The duration of study was from March to September 2015. IBBS surveys used respondent driven sampling (RDS) to select male IDUs in Kathmandu valley and Pokhara valley. Two stage cluster sampling was used to recruit male IDUs in Eastern Terai districts.

Face-to-face interviews were conducted by trained field workers in local language, Nepali, using a structured questionnaire. The independent variables for the study were socio-determinants (age, region, education, marital status), drug injecting practices (age of first drug injection, duration of drug use and injecting drugs, used needle/syringe previously used by someone else in past week, used syringe/needle left in a public place in past week, and shared needle/syringe with someone after using in past week) and sexual behaviors (age at first intercourse, number of sexual partners, number of FSWs) and consistent condom use with different sex partners. The sex partners were regular female sex partners, female sex workers (FSWs), and non-regular female sex partners. Regular female sex partner is spouse or any sexual partner living together with IDUs. FSWs were defined as those who sell sex in exchange for cash, kind, or drugs. Non-regular female sex partners were girlfriends or other female friends with whom IDUs have sexual relationship with but IDUs were neither married nor living together. The outcome variables for the study were HCV prevalence and HIV/HCV co-infection.

Informed consent was obtained from IDUs prior to the interview. There may be a risk of identifying the IDUs through their signatures if written consent was taken. The informed consent was taken in the presence of a witness (community motivators) who then signed the consent form. Study procedure was designed to protect participants’ privacy allowing for anonymous and voluntary participation. No names and personal identifiers were used in the data collection. Ethical clearances were permitted from Nepal Health Research Council.

Biological specimens were detected for HIV and HCV. Detection of HIV infection was carried out through rapid test kits following the HIV testing strategy II algorithm, which is based upon the “National Guidelines for Voluntary HIV and AIDS Counseling and Testing 2007”. HIV was detected through the use of ‘Determine HIV 1/2 (Abbott Japan Co. Ltd.)’ rapid test kits as a first test to detect antibodies against HIV. If the first test showed a negative result then no further tests were conducted. However, if the first test result was positive, the second test was performed using ‘Uni-Gold (Trinity Biotech, Dublin, Ireland)’ test kits. In cases of a tie between the first two tests, a third test was performed using ‘Stat Pak HIV 1/2 (Standard Diagnostics, Inc., Kyonggi-do, South Korea)’ as a tie-breaker test.

Dried blood spot samples were tested for the presence of antibodies against HCV by Blood/Serum/Plasma Dipstrip (Orgenics, Israel). The RapidSignal HCV whole blood/serum/plasma dipstrip kit was maintained under refrigeration. The kit components (dipstrips and buffer) were brought to room temperature. To begin the testing, the test dipstrip was removed from a sealed foil pouch. The tape was peeled off from the test stripe card and the dipstrip was stuck in the middle of the test card with arrows pointing down on the test card. Then, plasma sample was pipetted out and added onto the specimen pad of the dip strip. Then, two full drops of buffer were added and the timer was commenced. The sample was scored as reactive if two distinct red lines appeared- one in the control region and one in the test region. The sample was scored negative if the red line appeared only along the control line. If the red line did not appear at the control line irrespective of the test line, the test was considered invalid and the whole test was repeated using a new kit.

### Statistical analysis

Chi-squared test was used to examine the association of social determinants and risk behaviors with HCV prevalence and HIV/HCV co-infection. Logistic regression analyses were performed to determine factors associated with HCV and HIV/HCV co-infection defined by combinations of the determinants. First, the univariate logistic model was carried out among determinants that were significant in the Chi-squared test. All determinants significant with HCV and HIV/HCV co-infection prevalence in univariate logistic model were included the multivariate logistic model. This multivariate logistic model provides confidence intervals for outcomes for levels of each risk factor adjusted for other risk factors using sum contrasts methods. The confidence intervals based on sum contrast has an advantage that they provide a simple criterion for classifying levels of the factor into three groups according to whether each corresponding confidence interval exceeds, crosses, or is below the overall percent [20, 21]. The confidence intervals compared percent of the specified cause group in each category with the overall percent [22]. Receiver Operating Characteristic (ROC) curve was used to measure of goodness-of-fit and constructed mosaic plot. ROC plots sensitivity against the false positive rate to show how well a model predicts a binary outcome. It also gives mosaic plot for comparing logistic regression models similar to r-squared decomposition plot. R program was used for statistical analysis and creating graphs.

## Results

A total of 1045 male IDUs were included in the analysis, of whom 28.8% of them tested positive for HCV. Table 1 depicts the association between social determinants, drug injecting behaviours, sexual behaviours and HIV with HCV. Region, age, marital status, history of imprisonment, age of first drug initiation, duration of drug use and injecting drugs, shared needle/syringe with someone after using in the past week, consistent condom use with regular sexual partners, consistent condom use with non-regular sexual partners and presence of HIV were significantly associated with HCV (*p*-value <0.001). By region, Eastern Terai districts had the highest prevalence (47.5%) followed by Kathmandu Valley (22.1%) and Pokhara Valley (16%). HCV prevalence was found to be higher among older age groups; the prevalence being nearly half (45%) among 30–39 years. HCV among the married was 40%, higher than that of the unmarried population (22%). The majority of male IDUs (68%) was imprisoned or detained for any reasons and among them, HCV prevalence was 32%. Risky drug injecting behaviors such as longer duration of drug use and injecting drugs, used needle/ syringe previously used by someone, used syringe/needle left in public place and shared needle/syringe to someone after you using it were common in this population. IDUs who had first drug initiation above 30 years (41%) had higher HCV prevalence. HCV prevalence was higher among male IDUs whose duration of drug use and injecting drugs more than 10 years. IDUs who share needle/syringe to someone after used it in past week had higher HCV prevalence. IDUs who had consistent condom used with regular female sex partners had prevalence of 46% and inconsistent condom use with non-regular female sex partners had prevalence of 31%. Among those 6.2% of male IDUs who were HIV positive, co-infection with HCV was high, with 64.6% being co-infected.

**Table 1:** Association between social determinants, drug injecting behaviours, sexual behaviours and HIV with Hepatitis C

Characteristic	Total (*n* = 1045)	Hepatitis C (*n* = 301, 28.8%)	Not Hepatitis C (*n* = 744, 71.2%)	Χ^2^ (df)	*P*-value
	*n* (%)	*n* (%)	*n* (%)		
Region				96 (2)	< 0.001
Eastern Terai Region	360 (34.4)	171 (47.5)	189 (52.5)		
Kathmandu	340 (32.5)	75 (22.1)	265 (77.9)		
Pokhara	345 (33)	55 (15.9)	290 (84.1)		
Age				61 (2)	< 0.001
Below 20 years	210 (20.1)	31 (14.8)	179 (85.2)		
20–29 years	536 (51.3)	134 (25.0)	402 (75.0)		
30 years and above	299 (28.6)	136 (45.5)	163 (54.5)		
Education				3 (2)	0.17
No education	25 (2.4)	7 (28.0)	18 (72.0)		
Primary	141 (13.5)	50 (35.5)	91 (64.5)		
Secondary and above	879 (84.1)	244 (27.8)	635 (72.2)		
Marital status				40 (1)	< 0.001
Single	654 (62.6)	143 (21.9)	511 (78.1)		
Married	391 (37.4)	158 (40.4)	233 (59.6)		
History of Imprisonment				9.5 (1)	0.002
Yes	677 (64.7)	217 (32.1)	460 (67.9)		
No	368 (35.3)	84 (22.8)	284 (77.2)		
Age of drug initiation				6 (2)	0.03
Below 20 years	614 (58.8)	179 (29.2)	435 (70.8)		
20–29 years	360 (34.4)	93 (25.8)	267 (74.2)		
30 years and above	71 (6.8)	29 (40.8)	42 (59.2)		
Duration of drug use				85 (3)	< 0.001
Below 2 years	331 (31.7)	44 (13.3)	287 (86.7)		
2–10 years	446 (42.7)	129 (28.9)	317 (71.1)		
More than 10 years	268 (25.6)	128 (47.8)	140 (52.2)		
Duration of injecting drugs				113 (3)	< 0.001
Below 2 years	553 (53.0)	91 (16.5)	462 (83.5)		
2–10 years	356 (34.0)	129 (36.2)	227 (63.8)		
More than 10 years	136 (13.0)	81 (59.6)	55 (40.4)		
Used needle/ syringe previously used by someone in past week				2.1 (1)	0.157
Yes	40 (3.8)	16 (40)	24 (60)		
No	1005 (96.2)	285 (28.4)	720 (71.6)		
Use syringe/needle left in public place in past week				1.1 (1)	0.293
Yes	16 (1.5)	7 (43.8)	9 (56.2)		
No	1029(98.5)	294 (28.6)	735 (71.4)		
Share needle/syringe to someone after you used it in past week				6.9 (1)	0.009
Yes	47 (4.5)	22 (46.8)	25 (53.2)		
No	998(95.5)	279 (28)	719 (72)		
Age of first sex				2.5 (1)	0.117
Below 20 years	865 (82.7)	240 (27.7)	625 (72.3)		
Above 20 years	180 (17.3)	61 (33.9)	119 (66.1)		
Sexual contact with partners in past year				0.2 (1)	0.740
Yes	797 (76.3)	227 (28.5)	570 (71.5)		
No	248(23.7)	74 (29.8)	174 (70.2)		
Sexual contact with FSWs in past year				2.8 (1)	0.090
Yes	243(23.2)	59 (24.3)	184 (75.7)		
No	802(76.8)	242 (30.2)	560 (69.8)		
Consistent condom use with regular sexual partners				35 (1)	<0.001
Yes	210(20.1)	96 (45.7)	114 (54.3)		
No	835(79.9)	205 (24.6)	630 (75.4)		
Consistent condom use with FSWs				1.7 (1)	0.193
Yes	216(20.7)	54 (25)	162 (75)		
No	829(79.3)	247 (29.8)	582 (70.2)		
Consistent condom use with non-regular sexual partners					
Yes	359(34.3)	86 (24)	273 (76)	5.9 (1)	0.015
No	686(65.7)	215 (31.3)	471 (68.7)		
Consistent condom use with FSWs				1.7 (1)	0.193
Yes	216(20.7)	54 (25)	162 (75)		
No	829(79.3)	247 (29.8)	582 (70.2)		
HIV				41 (2)	< 0.001
Yes	65 (6.2)	42 (64.6)	23 (35.4)		
No	980 (93.8)	259 (26.4)	721 (73.6)		

HIV/HCV co-infection was prevalent in 4% of male IDUs. Region, age and duration of injecting drugs were associated with HIV/HCV co-infection (*p*-value <0.05), as shown in Table 2. Eastern Terai districts had the highest HIV/HCV co-infection (6.1%) compared to Kathmandu Valley (5.3%) and Pokhara Valley (0.6%). HIV/HCV co-infection was higher among older age groups (30 years and above) and longer duration of injecting drugs (more than 10 years).

**Table 2:** Association between social determinants, drug injecting behaviours, sexual behaviors with HIV/HCV co-infection

Characteristic	Total (*n* = 1045)	HIV/HCV co-infection (*n* = 42, 4%)	Not HIV/HCV co-infection (*n* = 1003, 96%)	Χ^2^ (df)	*P*-value
	n (%)	n (%)	n (%)		
Region				16 (2)	< 0.001
Eastern Terai districts	360 (34.4)	22 (6.1)	338 (93.9)		
Kathmandu	340 (32.5)	18 (5.3)	322 (94.7)		
Pokhara	345 (33)	2 (0.6)	343 (99.4)		
Age				20 (2)	< 0.001
Below 20 years	210 (20.1)	5 (2.4)	205 (97.6)		
20–29 years	536 (51.3)	12 (2.2)	524 (97.8)		
30 years and above	299 (28.6)	25 (8.4)	274 (91.6)		
Education				0.1 (2)	0.988
No education	25 (2.4)	1 (4)	24 (96)		
Primary	141 (13.5)	6 (4.3)	135 (95.7)		
Secondary and above	879 (84.1)	35 (4)	844 (96)		
Marital status				6 (1)	0.011
Single	654 (62.6)	18 (2.8)	636 (97.2)		
Married	391 (37.4)	24 (6.1)	367 (93.9)		
History of Imprisonment				2 (1)	0.157
Yes	677 (64.7)	10 (2.7)	358 (97.3)		
No	368 (35.3)	32 (4.7)	645 (95.3)		
Age of drug initiation				1.6 (2)	0.455
Below 20 years	614 (58.8)	27 (4.4)	587 (95.6)		
20–29 years	360 (34.4)	11 (3.1)	349 (96.9)		
30 years and above	71 (6.8)	4 (5.6)	67 (94.4)		
Duration of drug use				5 (2)	0.078
Below 2 years	331 (31.7)	10 (3)	321 (97)		
2–10 years	446 (42.7)	15 (3.4)	431 (96.6)		
More than 10 years	268 (25.6)	17 (6.3)	251 (93.7)		
Duration of injecting drugs				29 (2)	< 0.001
Below 2 years	553 (53.0)	15 (2.7)	538 (97.3)		
2–10 years	356 (34.0)	10 (2.8)	346 (97.2)		
More than 10 years	136 (13.0)	17 (12.5)	119 (87.5)		
Used needle/ syringe previously used by someone in past week				0.9 (1)	0.363
Yes	40 (3.8)	42 (4.2)	963 (95.8)		
No	1005 (96.2)	0 (0)	40 (100)		
Use syringe/needle left in public place in past week				1.2 (1)	0.272
Yes	16 (1.5)	40 (3.9)	989 (96.1)		
No	1029(98.5)	2 (12.5)	14 (87.5)		
Share needle/syringe to someone after you used it in past week				0.1 (1)	0.768
Yes	47 (4.5)	41 (4.1)	957 (95.9)		
No	998(95.5)	1 (2.1)	46 (97.9)		
Age of first sex				0.5 (1)	0.469
Below 20 years	865 (82.7)	37 (4.3)	828 (95.7)		
Above 20 years	180 (17.3)	5 (2.8)	175 (97.2)		
Sexual contact with partners in past year				0 (1)	1
Yes	797 (76.3)	10 (4)	238 (96)		
No	248(23.7)	32 (4)	765 (96)		
Sexual contact with FSWs in past year				0.1 (1)	0.921
Yes	243(23.2)	33 (4.1)	769 (95.9)		
No	802(76.8)	9 (3.7)	234 (96.3)		
Consistent condom use with regular sexual partners				2.5 (1)	0.111
Yes	210(20.1)	29 (3.5)	806 (96.5)		
No	835(79.9)	13 (6.2)	197 (93.8)		
Consistent condom use with FSWs				0 (1)	1
Yes	216(20.7)	33 (4)	796 (96)		
No	829(79.3)	9 (4.2)	207 (95.8)		
Consistent condom use with non-regular sexual partners				0.9 (1)	0.331
Yes	359(34.3)	31 (4.5)	655 (95.5)		
No	686(65.7)	11 (3.1)	348 (96.9)		

**Fig. 1:**
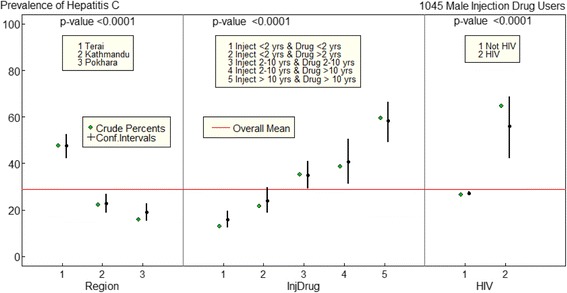
Factors associated with HCV prevalence in multivariate logistic regression

In multivariate logistic regression, there was statistically significant association between the presence of HCV and region, duration of drug use and injecting drugs, and the presence of HIV. However, there is no causal inference to show region is associated with HCV. The study found the interaction between duration of drug use and injecting drugs, so these determinants were combined. The number of levels of the Injecting-Drug group factor depends on duration of drug use and injecting drugs and there were five levels. The results from fitting logistic model for HCV prevalence were shown in Fig. 1, with region, duration of drug use and injecting drugs, and presence of HIV as determinants. Eastern Terai districts had the highest prevalence compared to Kathmandu Valley and Pokhara Valley of Nepal. Moreover, the HCV prevalence significantly increased with duration of drug use and injecting drugs. Higher prevalence was found among male IDUs who had longer duration of drug use and injecting drugs for more than 10 years. The presence of HIV was strongly correlated with HCV.

### Receiver Operating Characteristic (ROC) Curve

**Fig. 2:**
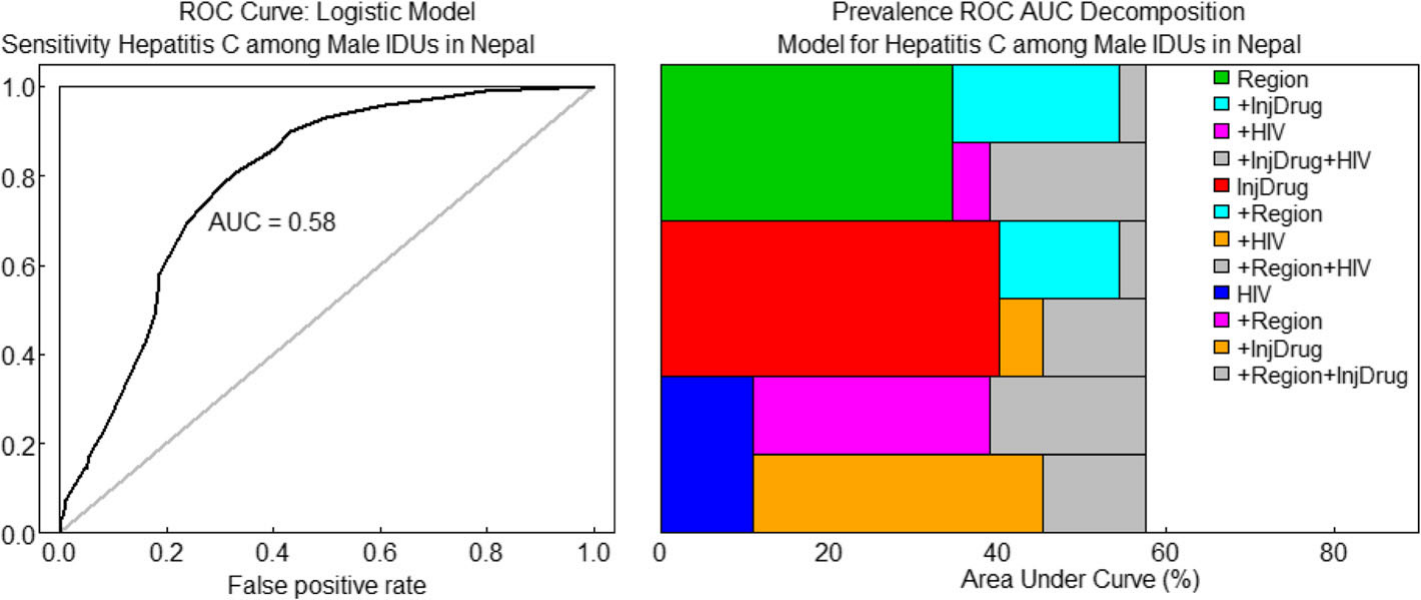
ROC curve: Logistic Model for HCV among male IDUs in Nepal. Notes: InjDrug is duration of drug use and injecting drugs

Figure 2 depicted ROC curves for the final model fitted to HCV prevalence. ROC curves provide the mosaic plot for comparing logistic regression models. The area under the curve (AUC) as 0.58, indicating model performance is fitted. ROC curves show that model containing region, duration of drug use and injecting drugs, and the presence of HIV as determinants fits the prevalence data extremely well.

**Fig. 3:**
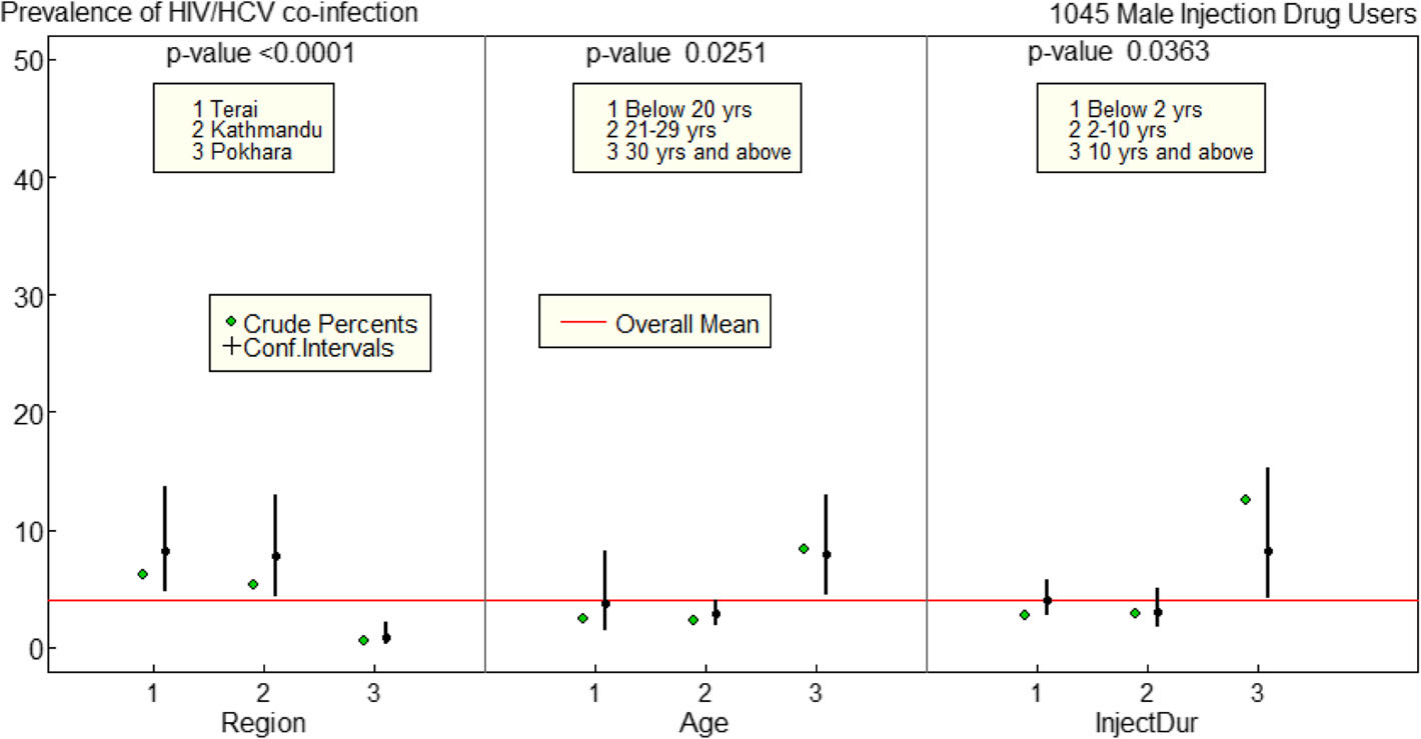
Factors associated with HIV/HCV co-infection in multivariate logistic regression

There was statistically significant association between the presence of HIV/HCV co-infection and age, region and duration of injection in multivariate logistic regression. Figure 3 shows results from fitting logistic model for HIV/HCV co-infection. Higher HIV/HCV co-infection was found in Eastern Terai districts, older age groups and had longer of injecting drugs.

**Fig. 4:**
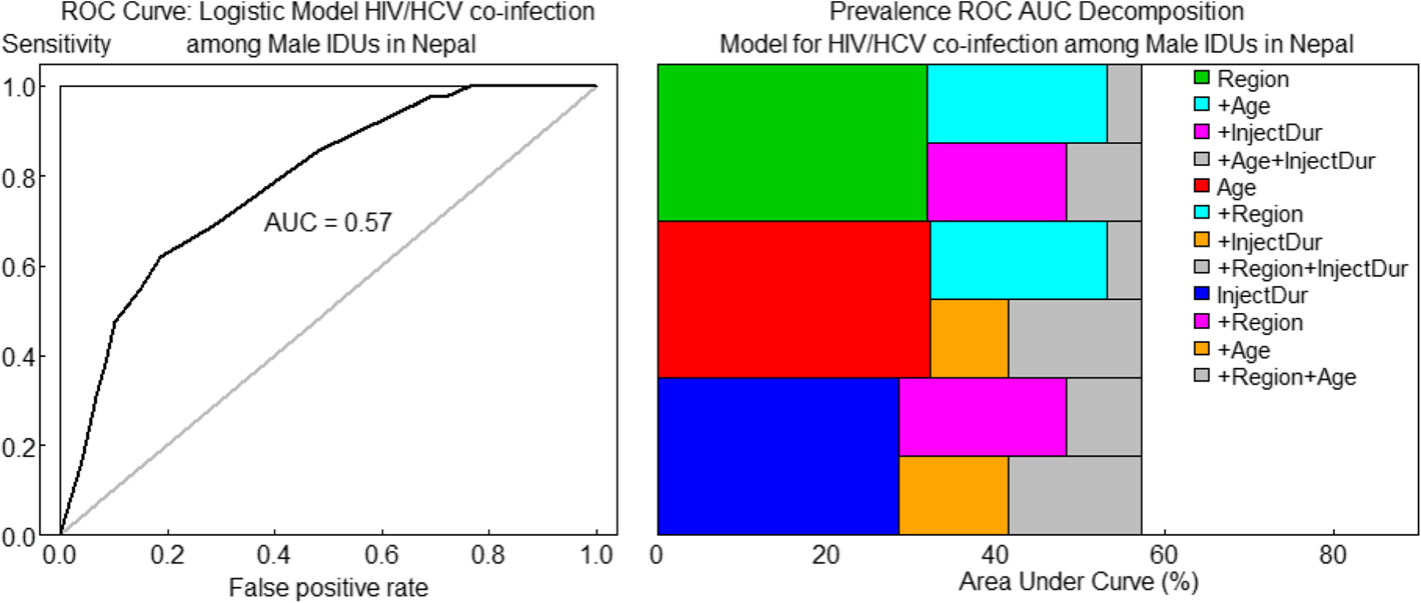
ROC curve: Logistic Model for HIV/HCV co-infection among male IDUs in Nepal. Notes: InjDrug is duration of injecting drugs

Figure 4 showed ROC curves for the final model fitted to HIV/HCV co-infection. The area under the curve (AUC) as 0.57, indicating model performance is fitted.

## Discussion

This study showed HCV infection was prevalent among male IDUs; however it was lower than recent study in 2015 that showed HCV prevalence of 41.9% [13]. The major concern was the high prevalence of HCV infection among those who were HIV infected (65%), highlighting the importance of testing all HIV infected male IDUs for HCV before commencing ART.

There existed marked variation of HCV and HIV/HCV co-infection, as HCV and HIV/HCV prevalence was higher in Eastern Terai districts compared to Kathmandu Valley and Pokhara Valley. In Nepal, IDUs are confined in urban metropolises and eastern highway districts [23]. The territories of the Terai area are bordered to India and became a principal transit point for peddling drugs and provided an ideal passage for smugglers for decades [24]. Moreover, IBBS reports suggest that male IDUs in eastern highway districts are often poor, uneducated, came from rural areas, possessed unsafe injecting behaviors, had unprotected sexual contacts with sex workers and lacked access to local HIV/STI intervention compared to their counterparts in urban cities [25–28].

Old age was associated with HCV infection in bivariate analysis and associated with HIV/HCV co-infection in multivariate analysis. These results corroborate the outcomes of other studies demonstrating the increase of the HCV prevalence as age increases [6, 13]. The prevalence of HCV and HIV/HCV co-infection was found among aged 30 years and above. A recent study in Nepal showed that HCV prevalence correlated significantly with age [13]. A similar study in Brazil found that the HCV prevalence was highest over 30 years old, with a rise in patients aged 50 to 59 years (3.8%) [29]. Moreover, the study also showed HCV infection was prevalent above 40 years old in Malaysia [30].

Moreover, HCV infection rates were higher among married IDUs. In the context of drug injecting practices, IDUs who had HCV can transmit the virus to their wives and other sexual partners. The combination of HCV, drug use and sexual behaviors make them at high risk and an important source of infection to others. These IDUs should be reached by program and treated in order to prevent transmission to different sexual partners.

Imprisonment history was predictor for HCV infection in our study but these associations did not remain significant in the final multivariate analysis. These results were in accordance with studies showing HCV prevalence was higher among prisoners and ex-convicts [31, 32]. Studies reported high risk behaviors like drug abuse inside or outside the prisoners, which predispose the prisoners to infections of hepatitis B, C, and HIV [30, 31].

Consistent with previous studies, the established risk factors of duration of injection use were strongly related to HCV infections [4, 6–9]. Longer duration of drug use and injecting drugs significantly increase risk for HCV and HIV/HCV co-infection, and in a dose-dependent manner. The longer the IDUs are exposed to drug injecting behaviours, the more they are expose to greater numbers of injecting partners. The higher the concurrency of these partners, the more risk they are expose. Moreover, they are also more likely to engage in harmful alcohol use, substance abuse, sexual behaviours and contracting HIV infection when contact with a large number of partners [4, 13–16].

Unsafe injecting behaviors are the most common drug related risk behaviors, which puts the IDUs at risk of HCV transmission. In our study, sharing needle/syringe to someone after using in past week was significantly associated with HCV prevalence in bivariate analysis. However, in multivariate analysis, no significant association was found. Previous studies delineated that unsafe injecting behaviour while preparing drug and practices of drug sharing among users (splitting drugs prepared by a user with subsequent transport of the prepared drug from one syringe to other syringe, sharing cotton, filters, cooker, water and water containers) were associated with HCV transmission [1, 2, 14–17].

In addition to this, sexual behaviour was also leading risk factor for HCV transmission. The risk significantly increased with non-regular female sex partners for unprotected sex. IDUs perceive the risk of transmission is less likely through sexual contact than through drug injecting behaviours which results IDUs to adopt unsafe sex behaviour than safe injection practices. In contrast, the study also found that HCV was higher among IDUs who had consistent condom use with regular female sex partners. IDUs may be aware of their status and were using condoms to either avoid HCV transmission to their partners, or as self-protection from re-infection.

HIV was strongly correlated with HCV as 65% of male IDUs in the study were found to be HCV among HIV infected. HCV is increasing reported to be leading cause of deaths among HIV infected [1, 2, 4, 13–16]. This double burden of HCV and HIV among male IDUs need to be controlled to prevent further transmission.

As IBBS survey is cross-sectional by their intent and providence of causal relationships between the determinants and outcome is constraint in this study. This study covered social determinants and risk behaviors, while other issues related to intervention programs and drug policies were not analyzed in detail, and suggest for further studies.

## Conclusion

HCV was highly prevalent among male IDUs. Injection drug use behaviors remain independent risk factors for HCV infection. IDUs therefore constitute an important source of viral infections and therefore can play a mediator for the transmission of viruses to the general population. Various intervention programs already being carried out by Nepal government for HIV related service delivery needs to be extended towards HCV infected population for the prevention of HCV transmission. Moreover, focussed health interventions are needed in Eastern Terai districts and IDUs with longer duration of injecting behaviours.

## Abbreviations

FSW: Female Sex Workers
HCV: Hepatitis C Virus
HIV: Human Immunodeficiency Virus
IBBS: Integrated Biological and Behavioural Surveillance
MALE IDU: Male Injecting Drug Users
NCASC: National Centre for AIDS and STD Control
ROC: Receiver operating characteristic
STI: Sexually Transmitted Infections
